# Comparison of 16S rRNA gene amplicon and whole-genome shotgun metagenomic sequencing for subgingival oral microbiome profiling

**DOI:** 10.1080/20002297.2026.2679807

**Published:** 2026-05-27

**Authors:** Jung Hwa Park, Jin Chung, Hyo-Jung Lee, Hee Sam Na

**Affiliations:** a Department of Oral Microbiology, School of Dentistry, Pusan National University, Yangsan, Republic of Korea; b Department of Oral Microbiology, School of Dentistry, Chonnam National University, Gwangju, Republic of Korea; c Department of Periodontology, Section of Dentistry, Seoul National University Bundang Hospital, Seongnam, Republic of Korea; d Department of Microbiology and Immunology, Chonnam National University Medical School, Hwasun, Jeollanam-do, Republic of Korea

**Keywords:** Periodontitis, subgingival plaque, oral microbiome, 16S rRNA gene sequencing, whole-genome shotgun sequencing

## Abstract

**Background:**

Periodontitis is a chronic inflammatory disease driven by a dysbiotic subgingival microbiome. While 16S rRNA gene amplicon sequencing is widely used, whole-genome shotgun (WGS) metagenomics is increasingly applied for higher taxonomic and functional resolution.

**Objective:**

The aim of this study was to directly compare 16S rRNA gene amplicon (V1–V2) sequencing and WGS metagenomic sequencing using matched subgingival plaque samples from patients with periodontitis.

**Methods:**

Subgingival plaque samples from 28 patients with periodontitis were analyzed using both 16S rRNA gene amplicon (V1–V2) sequencing and WGS metagenomics. Taxonomic composition, microbial diversity, differential abundance and functional analysis were compared across platforms.

**Results:**

WGS generated markedly higher read counts than 16S rRNA gene amplicon but showed wide variability in non-human reads, whereas 16S rRNA gene amplicon yielded a consistent proportion of non-chimeric reads. High taxonomic overlap was observed at the phylum level but declined at higher taxonomic ranks. WGS preferentially detected taxa such as *Actinomyces*, *Corynebacterium* and *Olsenella*, while the 16S rRNA gene amplicon more frequently captured *Saccharibacteria* (TM7) and low-abundance taxa. Core genera, including *Rothia*, *Neisseria* and *Cardiobacterium* showed comparable abundance patterns across platforms. When patients were grouped depending on probing pocket depth (PPD), LEfSe analysis resulted in platform-specific enrichment patterns. Functional analyses revealed shared central pathways, such as pyruvate metabolism, while 16S-based PICRUSt2 emphasized reductive and degradative pathways and WGS-based HUMAnN highlighted oxidative and biosynthetic pathways. Notably, WGS-based functional profiles were strongly influenced by microbial read depth.

**Conclusions:**

This comparative analysis demonstrates that 16S rRNA gene amplicon (V1–V2) sequencing and WGS both robustly capture core subgingival microbial signatures. While WGS provides higher species-level and functional resolution, the resolution was strongly constrained by microbial read depth in host-rich subgingival samples. These findings provide practical guidance for selecting appropriate sequencing strategies and optimizing sample preparation when designing WGS-based periodontal microbiome studies.

## Introduction

The human oral cavity harbours a diverse and complex microbial community, which plays a critical role in maintaining oral and systemic health [1,2]. Periodontitis is a chronic inflammatory disease driven by dysbiosis of the dental plaque biofilm and a dysregulated host immune response [3,4]. Recent clinical and experimental studies have increasingly emphasized that therapeutic interventions for periodontitis, including different supportive periodontal care protocols and antimicrobial formulations, influence clinical outcomes and are accompanied by changes in the oral microbial community. This highlights the importance of accurately characterizing microbial community dynamics in both disease and health maintenance [5,6]. To investigate the composition and function of the oral microbiome, various sequencing-based approaches have been employed. Among these, 16S ribosomal RNA (rRNA) gene amplicon sequencing and whole-genome shotgun (WGS) metagenomic sequencing are the most widely used, differing substantially in methodology, resolution and functional capacity [7–10].

16S rRNA gene amplicon sequencing targets hypervariable regions of the bacterial 16S rRNA gene and is widely used owing to its cost-effectiveness, high-throughput capability and well-established analytical pipelines [11]. However, its ability to distinguish closely related species is limited, particularly among taxa with highly conserved 16S rRNA gene sequences [12–14]. In addition, 16S rRNA gene amplicon sequencing does not capture non-bacterial taxa such as fungi, viruses, or archaea, and it lacks direct access to functions related to microbial metabolism, virulence, or antibiotic resistance [15]. Consequently, this approach has been widely used in large-scale microbial diversity surveys where broad taxonomic resolution is sufficient. It has been extensively applied in studies investigating the human microbiota, including the gut, oral cavity, and skin, as well as in comparative analyses of microbial communities in soil and marine ecosystems [16–19].

In contrast, WGS involves random fragmentation and sequencing of all genomic DNA present in a sample. This approach enables comprehensive taxonomic profiling, including bacteria, archaea, fungi and viruses, and facilitates functional characterization at the gene and metabolic pathway levels [20,21]. Moreover, WGS offers higher taxonomic resolution, allowing identification at the species and even strain level, which is critical for understanding fine-scale microbial diversity [22]. However, WGS requires high-performance computing resources and careful preprocessing to remove host DNA contamination, especially for human-related samples such as saliva or plaque, where host reads can account for more than 90% of the total [23,24]. Accordingly, WGS is typically applied in studies requiring detailed community structure, functional profiling, genome assembly and microbial contributions to health and disease [22,25].

Comparing 16S rRNA gene amplicon and WGS metagenomic sequencing, therefore, enables evaluation of the strengths and limitations of each approach, validation of cross-platform microbial signatures, and guidance for methodological decisions in future microbiome studies. Several studies have compared these two sequencing platforms in the context of human microbiome research, particularly in gut samples. These studies have provided insights into the differences in taxonomic resolution, diversity estimation and functional prediction between the two platforms [15,26–29]. In oral microbiome research, 16S rRNA gene amplicon sequencing has been predominantly employed because of its cost-effectiveness and established workflows, providing valuable insights into the microbial diversity of saliva, dental plaque and other oral niches [30–32]. However, WGS has been increasingly adopted, allowing deeper investigation of community composition and functional potential. For instance, recent investigations have employed WGS to define the complexity of the healthy oral microbiome and its resistome [33], to characterize microbial alterations associated with oral cavity squamous cell carcinoma [34], and to explore potential microbial factors linked to chronic bacterial osteomyelitis [35]. Recently, oral microbiome research has expanded beyond taxonomic profiling to include functional analyses that explore microbial metabolism, virulence and ecological interactions [36–38]. Nonetheless, the two sequencing platforms differ in their analytical principles and depth of information, often leading to inconsistencies in taxonomic resolution and functional interpretation across studies [28,39,40]. In this context, studies directly comparing 16S rRNA gene amplicon and WGS metagenomic sequencing using identical samples are needed to assess the consistency of the analytical outcomes. However, such comparative studies focusing on the oral microbiome remain limited.

Among the various oral microbial habitats, the subgingival compartment has been recognized as a key ecological niche in periodontitis pathogenesis [3,4]. The subgingival niche provides an anaerobic, protein-rich environment that supports colonization by fastidious Gram-negative bacteria [41,42]. The subgingival microbiota plays a central role in periodontitis by shifting from a symbiotic to a dysbiotic community, enabling immune subversion, driving chronic inflammation, and contributing to connective tissue and bone destruction [3,43]. Studying the subgingival microbiome is uniquely challenging owing to the limited raw biomass, high levels of host DNA contamination, strong site-to-site heterogeneity, and the dominance of anaerobic, disease-associated taxa. These features make it distinct from other oral niches (saliva, supragingival plaque and buccal mucosa) and gut microbiome studies, where the biomass is larger, contamination is lower, and community structure is more homogeneous [4,41,44]. Although many studies target the V3–V4 16S rRNA region, several reports indicate that the V1–V2 region provides taxonomic coverage comparable to that of V3–V4 primers, while offering improved species-level resolution after paired-end read merging in oral microbiome studies [14,45]. However, direct comparisons between V1–V2 amplicon datasets and whole-genome shotgun sequencing remain scarce.

Therefore, there is a clear need to systematically and clinically evaluate how the choice of sequencing platform influences the interpretation of the subgingival microbiome in periodontitis, a niche that plays a central role in disease pathogenesis and therapeutic monitoring. In this study, we performed a direct comparison of 16S rRNA gene amplicon (V1–V2 region) and WGS sequencing using matched subgingival plaque samples from patients with periodontitis. The aim of this study was to comprehensively evaluate platform-dependent differences in sequencing output, microbial diversity, taxonomic resolution, and functional profiling, and to provide practical guidance for selecting appropriate sequencing strategies in periodontal microbiome research.

## Materials and methods

### Study population and sample collection

The periodontitis group included patients diagnosed with chronic periodontitis based on clinical examination and radiographic assessment. The exclusion criteria included antibiotic use within the past month, periodontal treatment within the past 6 months, the use of immunosuppressants or corticosteroids, uncontrolled diabetes, severe systemic diseases, and refusal to provide informed consent. All participants were fully informed of the study purpose and procedures and provided written consent prior to enrollment. This study was approved by the Institutional Review Board of Seoul National University Dental Hospital (IRB No. B-2004-604-301, B-2001-586-303, B-2104-680-301) and conducted in accordance with the Declaration of Helsinki [46].

All participants attended a single study visit during which both oral sample collection and periodontal clinical examination were performed. Subgingival plaque samples were obtained from periodontal pockets with a single-stroke curette method. Alongside sample collection, a comprehensive periodontal examination was conducted to record the probing pocket depth (PPD), clinical attachment level, plaque index, gingival index, bleeding on probing, gingival recession and the DMFT index.

### Extraction of genomic DNA and next-generation sequencing

Total DNA was extracted from the subgingival plaque using a Gram-positive DNA purification kit (Lucigen, Biosearch Technology, Novato, CA) following the manufacturer’s instructions. For 16S rRNA gene amplicon sequencing, each sequenced sample was prepared according to the Illumina 16S Metagenomic Sequencing Library protocols to amplify the V1 and V2 region (27F-338R). The barcoded fusion primer sequences used for amplifications were as follows: 27F: 5’-AGA GTT TGA TYM TGG CTC AG-3’, 338R: 5’-TGC TGC CTC CCG TAG RAG T-3’ [14]. For WGS, the sequencing libraries were prepared according to the manufacturer’s instructions of TruSeq Nano DNA High Throughput Library Prep Kit (Illumina). Briefly, 200 ng of genomic DNA was sheared using adaptive focused acoustic technology (Covaris) and the fragmented DNA was end-repaired to create 5’-phosphorylated, blunt-ended dsDNA molecules. Following end-repair, DNA was size-selected with a bead-based method. These DNA fragments go through the addition of a single ‘A’ base and ligation of the TruSeq DNA UD Indexing adapters. The products are then purified and enriched with PCR to create the final DNA library.

The libraries were quantified using KAPA Library Quantification kits for Illumina Sequencing platforms according to the qPCR Quantification Protocol Guide (KAPA BIOSYSTEMS, #KK4854) and qualified using the TapeStation D1000 ScreenTape (Agilent Technologies, # 5067-5582). Indexed libraries were then submitted to an Illumina NovaSeq6000 (Illumina, Inc., San Diego, CA, USA), and the paired-end (250 × 2 bp) sequencing was performed by Macrogen Incorporated.

### Bioinformatic analysis, statistical analysis and visualization

For 16S rRNA gene amplicon sequencing analysis, all bioinformatic processing was conducted in QIIME2 (v2024.10) using standard QIIME2 plugins [47]. Paired-end FASTQ reads (Phred33) were imported, and the demultiplexed read quality was assessed to guide downstream trimming and truncation settings. Denoising and paired-end read merging were performed with the QIIME2 DADA2 plugin. Forward and reverse reads were trimmed by 8 bp from the 5′ end and truncated at 245 bp (forward) and 240 bp (reverse). The denoising step produced an amplicon sequence variant (ASV) feature table and representative ASV sequences. Denoising statistics were used to summarize preprocessing results. Taxonomic assignment of ASVs was performed using a pre-trained Naive Bayes classifier trained on the Human Oral Microbiome Database (eHOMD) 16S rRNA Extended RefSeq sequences (v15.1) [40], using the default confidence threshold implemented in QIIME2 (confidence = 0.7). Metagenome functional potential was inferred from 16S rRNA gene ASVs using PICRUSt2 within QIIME2 [48]. The denoised ASV feature table and representative ASV sequences were provided as inputs to `qiime picrust2 full-pipeline`, which performs hidden-state prediction and generates predicted gene-family and pathway profiles. The predicted pathway abundances and KEGG Ortholog (KO) metagenome predictions were then summarized in QIIME2. Preprocessing information is shown in Supplementary Table 1.

For whole-genome shotgun (WGS) metagenomic data analysis, paired-end FASTQ reads were preprocessed, host-depleted, and profiled for taxonomy and function. The raw reads were quality filtered with Trimmomatic in paired-end mode (Phred33). Adapter trimming used the TruSeq3 paired-end adapter set with ‘ILLUMINACLIP:2:30:10’, and additional quality filters were applied (‘LEADING:3’, ‘TRAILING:3’, ‘SLIDINGWINDOW:4:20’, ‘MINLEN:36’). To remove human-derived sequences, the trimmed paired reads were aligned to a pre-built human reference index (GRCh38(hg38)) using Bowtie2 with ‘--very-sensitive’. Host-unmapped paired reads were extracted using `samtools view -f 12 -F 256`, sorted by read name, and converted back to paired FASTQ using ‘samtools fastq’ to generate host-depleted read pairs. Taxonomic profiling was performed on host-depleted paired reads using Kraken2 with a prebuilt standard reference database, generating a classification output. Species-level abundance estimates were refined using Bracken with read length set to 100 bp and the taxonomy level set to species. Preprocessing metrics and read-retention statistics from these steps were compiled and are summarized in Supplementary Table 2. Functional profiling was conducted using HUMAnN (v3.6) on host-depleted reads [49]. For each sample, HUMAnN was executed using a nucleotide database (ChocoPhlAn) and a protein database (UniRef). Gene family outputs were subsequently regrouped to KEGG Orthologs (KOs). Group differences in pathway or metabolite-potential abundances were assessed with DESeq2, and significant pathways were visualized with clusterProfiler v.4.8.3.

Microbiome analysis was conducted by phyloseq (1.46.0) and related packages in R software v4.3.1. To measure alpha diversities, the Chao1 index and the Shannon index were used. Beta diversity was assessed using Aitchison distances calculated as Euclidean distances on centred log-ratio transformed compositional abundance data. Prior to centered log-ratio (CLR) transformation, zero counts were replaced by adding a pseudocount of 0.5, and the count table was converted to relative abundances. Principal component analysis (PCA) was performed on the CLR-transformed abundance matrix. Differences in community composition among groups were tested using PERMANOVA with 999 permutations, followed by pairwise PERMANOVA with Benjamini–Hochberg correction. To evaluate the global concordance of microbial community structures between the 16S rRNA gene amplicon and WGS datasets, the *Procrustes* function in the *vegan* R package was performed using matched sample ordination coordinates [50]. The analysis was conducted at both the genus and species levels. Statistical significance was assessed by permutation testing with 9,999 permutations. To test the differential abundance of bacterial species among the groups, linear discriminant analysis effect size (LEfSe) [51] was applied with the default settings.

## Results

### Participants characterization

A total of 28 participants with periodontitis were enrolled in this study. For each participant, oral samples were collected from the subgingival plaque, which were analyzed using both 16S rRNA gene amplicon and WGS metagenomic sequencing. Detailed participant information is presented in Table 1. The participants were further classified into two groups based on their PPD: shallow pocket (≤3 mm) and deep pocket (>3 mm).

**Table 1.** Characterization of patients (mean ± SD).

Variables	Shallow pocket (*n* = 10)	Deep pocket (*n* = 18)	*P-*value	
Age	46.1 ± 15.8	54.9 ± 14.1	0.161	
Sex (M/F)	2/8	12/6	0.018	
Height	171.1 (3.2)	170.8 (10.4)	0.811	
Weight	96.2 (28.6)	74.3 (22.3)	0.015	
**Periodontal clinical parameters**				
PPD (mm)	2.5 (0.3)	3.4 (1.6)	0.011	
CAL (mm)	3.4 (0.2)	3.8 (1.1)	0.262	
GI	0.3 (0.8)	0.4 (0.9)	0.390	
PI	0.5 (0.5)	0.8 (0.3)	0.116	

Abbreviations: PPD, probing pocket depth; CAL, clinical attachment level; GI, gingival index; PI, plaque index.

^*^Periodontitis severity was assessed by dental experts and classified according to the Centers for Disease Control and Prevention-American Academy of Periodontology (CDC-AAP) definition.

### Read counts during preprocessing

The total read counts obtained from WGS (35,408,562 ± 4,511,727) were significantly higher than those from 16S rRNA gene amplicon sequencing (240,352 ± 118,580). For 16S rRNA gene amplicon sequencing, the final non-chimeric read count was 75,999 ± 36,045, and the percentage of non-chimeric reads relative to input reads was 31.9 ± 2.7% (Supplementary Table 1). In contrast, the final unmapped read count for WGS was 19,337,348 ± 13,105,860, with the percentage of unmapped reads relative to input reads at 54.0 ± 35.3% (Supplementary Table 2). While 16S rRNA gene amplicon sequencing showed a relatively consistent percentage of non-chimeric reads (ranging from 25.8% to 36.46%), WGS showed a wide range in the percentage of unmapped reads (non-human reads), from 2.75% to 91.91%. Taken together, the higher number of input reads in the WGS data mainly reflects differences in sequencing design, whereas 16S rRNA gene amplicon sequencing provided a more stable proportion of non-chimeric reads.

### Taxonomic composition at the phylum level across platforms

**Figure 1.**
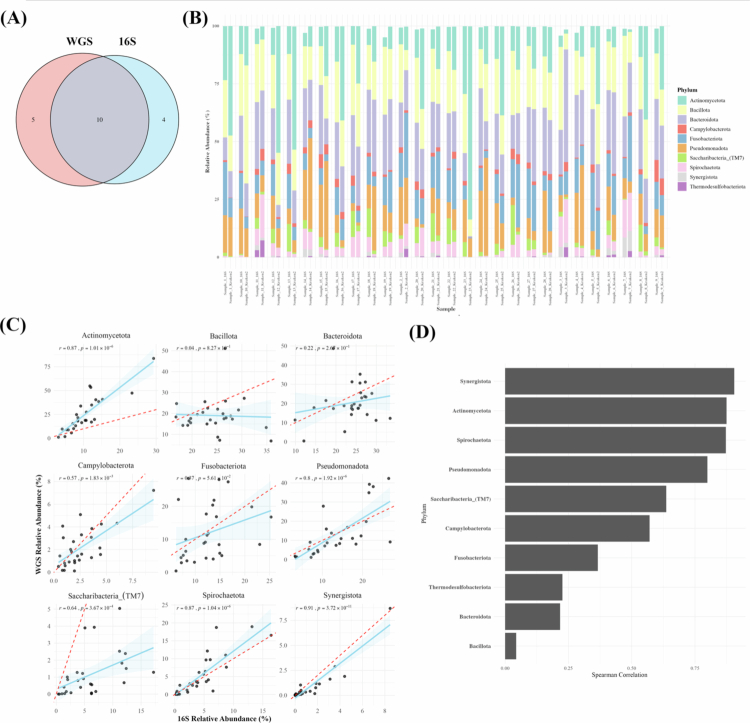
Comparison on relative abundance depending on platforms at phylum level. (A) Number of phyla found depending on platforms, (B) individual sample of relative abundance of top 10 phyla found in both platforms, (C) correlation of relative abundance of top 9 phyla and (D) summary of phylum correlation. In the correlation plots, each point represents one matched sample. The x-axis indicates the relative abundance estimated from the 16S rRNA gene amplicon dataset, and the y-axis indicates the corresponding relative abundance estimated from the WGS dataset. The solid blue line represents the fitted correlation trend across samples, whereas the dotted red line indicates the 1:1 line of absolute agreement between the two platforms.

To compare taxonomic resolution at the phylum level between the two sequencing platforms, we assessed the number of detected phyla, their relative abundance, and inter-platform correlations. Phyla less than 0.01% were removed before analysis. A total of 15 phyla were identified in the 16S dataset, and 14 phyla in the WGS dataset. Among these, 10 phyla were commonly detected in both platforms, accounting for approximately 67% overlap (Figure 1A). The relative abundance of the top 10 phyla identified by each platform across all samples is shown in Figure 1B. While several phyla showed broadly similar abundance trends, phyla such as Actinomycetota and Saccharibacteria (TM7) showed noticeable differences in relative abundance.

To assess the concordance in taxonomic profiling between the two sequencing platforms, we compared the relative abundance of the top 9 commonly detected phyla using correlation analysis (Figure 1C). In these scatter plots, the x-axis represents the abundance values derived from the 16S dataset, while the y-axis indicates those from the corresponding WGS dataset. The red dashed line represents the 1:1 line of absolute agreement, and the blue line indicates the actual correlation trend across samples. Actinomycetota consistently showed higher relative abundance in the WGS dataset, whereas Saccharibacteria (TM7) was more abundantly detected in the 16S dataset (Figure 1C). Individual abundance correlation for the top phyla is summarized in Figure 1D. Several phyla, including Synergistota, Spirochaetota and Actinomycetota, showed strong correlations in their abundance patterns across platforms. In contrast, phyla such as Bacillota and Bacteroidota showed weak correlations.

### Taxonomic composition at the genus level across platforms

**Figure 2.**
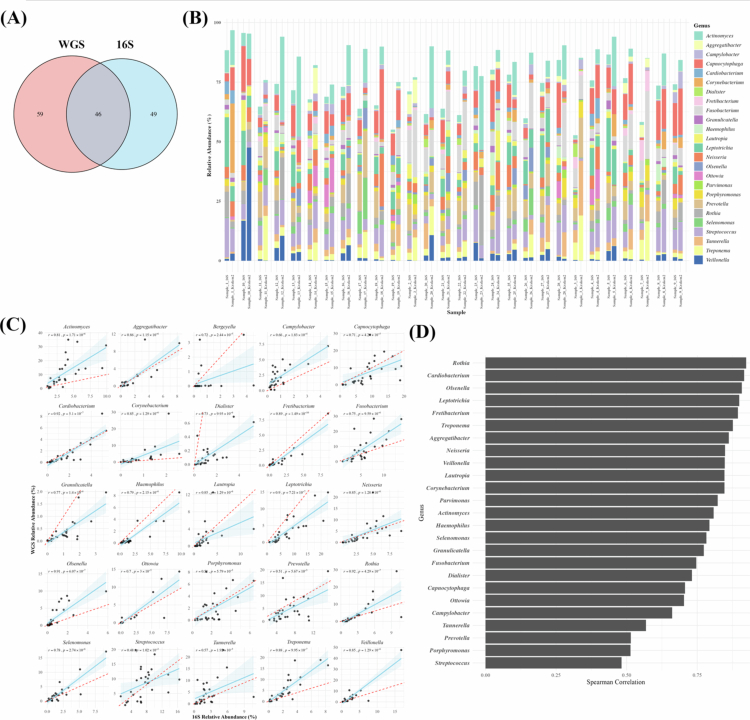
Comparison of relative abundance depending on platforms at the genus level. (A) Number of genus found depending on platforms, (B) individual sample of relative abundance of top 25 genus found in both platforms, (C) correlation of relative abundance of top 25 genus and (D) summary of genus correlation. In the correlation plots, each point represents one matched sample. The x-axis indicates the relative abundance estimated from the 16S rRNA gene amplicon dataset, and the y-axis indicates the corresponding relative abundance estimated from the WGS dataset. The solid blue line represents the fitted correlation trend across samples, whereas the dotted red line indicates the 1:1 line of absolute agreement between the two platforms.

At the genus level, a total of 95 genera were identified in the 16S dataset and 105 genera in the WGS dataset. Among these, 46 genera were commonly detected across both platforms, accounting for approximately 47% overlap (Figure 2A). The relative abundance of the top 25 genera identified by each platform across all samples is shown in Figure 2B. While several genera, including *Aggregatibacter*, *Cardiobacterium*, *Fretibacterium*, *Neisseria* and *Rothia*, exhibited comparable abundance patterns between the two platforms, others showed differential distribution.

To evaluate the consistency of genus-level taxonomic profiles between the two sequencing platforms, we performed correlation analysis on the top 25 genera commonly identified across datasets (Figure 2C). Notably, *Actinomyces*, *Corynebacterium* and *Olsenella* showed consistently higher relative abundance estimated in the WGS dataset, while *Bergeyella*, *Dialister* and *Granulicatella* were more predominant in the 16S dataset. The individual abundance correlation for the top genus is summarized in Figure 2D. Several genera, including *Rothia, Cardiobacterium*, *Olsenella, Leptotrichia* and *Treponema* showed strong correlations in their abundance patterns across platforms. In contrast, genus such as *Streptococcus, Porphyromonas* and *Prevotella* showed weak correlations.

### Taxonomic composition at the species level across platforms

**Figure 3.**
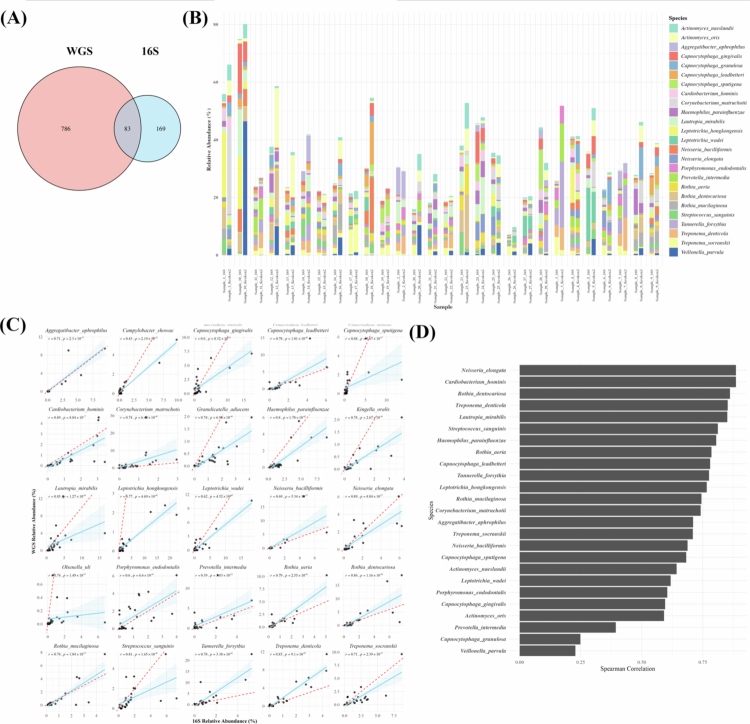
Comparison of relative abundance depending on platforms at the species level. (A) Number of species found depending on platforms, (B) individual sample of relative abundance of top 25 species found in both platforms, (C) correlation of relative abundance of top 25 species and (D) summary of species correlation. In the correlation plots, each point represents one matched sample. The x-axis indicates the relative abundance estimated from the 16S rRNA gene amplicon dataset, and the y-axis indicates the corresponding relative abundance estimated from the WGS dataset. The solid blue line represents the fitted correlation trend across samples, whereas the dotted red line indicates the 1:1 line of absolute agreement between the two platforms.

At the species level, a total of 252 species were identified in the 16S dataset and 869 species in the WGS dataset. Among these, 83 species were commonly detected across both platforms, corresponding to less than 35% overlap based on the 16S dataset. When calculated based on the WGS dataset, the overlap accounts for approximately 9.6% (Figure 3A). The relative abundance of the top 25 species identified by each platform across all samples is shown in Figure 3B. While several species, such as *Aggregatibacter aphrophilus*, *Porphyromonas endodontalis*, *Prevotella intermedia* and *Rothia mucilaginosa*, showed broadly similar abundance patterns between platforms, several other taxa showed variations in distribution between the two sequencing platforms.

To evaluate the consistency of species-level taxonomic profiles between the two sequencing platforms, we performed correlation analysis on the top 25 genera in the two datasets (Figure 3C). Especially, *Corynebacterium matruchotii*, *Tannerella forsythia* and *Treponema denticola* were consistently more abundant in the WGS dataset, whereas *Granulicatella adiacens*, *Kingella oralis* and *Olsenella uli* were more abundantly represented in the 16S dataset. The individual abundance correlation for top species is summarized in Figure 3D. Several species, including *Neisseria elongata, Cardiobacterium hominis, Rothia denticariosa, T. denticola* and *Lautropia mirabilis,* showed strong correlations in their abundance patterns across platforms. In contrast, genus such as *Veillonella parvula, Capnocytophaga granulosa* and *Prevotella intermedia* showed weak correlations.

### Microbial diversity differences between 16S and WGS platforms

**Figure 4.**
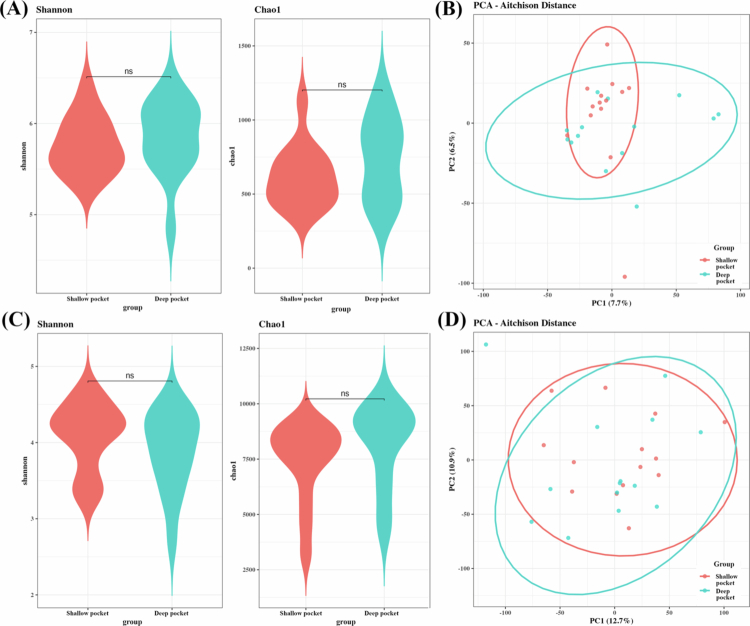
Bacterial community comparisons depending on platforms. (A) 16S alpha diversity, (B) 16S beta diversity, (C) WGS alpha diversity and (D) WGS beta diversity. Alpha diversity was used to describe the microbial richness and evenness within samples using the Chao1 and Shannon indices. For beta diversity, abundance profiles were transformed using the CLR transformation, and Aitchison distances were calculated as Euclidean distances on the CLR-transformed data. PCA was performed to evaluate the microbial community structure.

To evaluate differences in microbial diversity between sequencing platforms, the alpha diversity was assessed using the Chao1 and Shannon indices. Both 16S and WGS datasets showed no significant differences in alpha diversity between the shallow pocket and deep pocket (Figure 4A, C). Beta-diversity was evaluated using a compositionality-aware analysis based on the Aitchison distance after CLR transformation. In the 16S rRNA gene amplicon dataset, the original analysis showed no significant difference in microbial community composition between the shallow pocket and deep pocket (F = 1.0863, R² = 0.0401, *p* = 0.204, BH-adjusted *p* = 0.204). Consistently, the Aitchison distance-based PERMANOVA also showed no significant group-level difference (F = 1.0892, R² = 0.0402, *p* = 0.115; BH-adjusted *p* = 0.117). Similarly, in the WGS dataset, the original analysis was not significant (F = 1.3062, R² = 0.0478, *p* = 0.165, BH-adjusted *p* = 0.165), and the Aitchison distance-based analysis also showed no significant separation between groups (F = 1.0789, R² = 0.0398, *p* = 0.270; BH-adjusted *p* = 0.326) (Figure 4B, C). These results indicate that the overall microbial community structure did not differ significantly between the shallow pocket and deep pocket in either 16S rRNA gene amplicon or WGS profiles, and this conclusion was robust to the use of Aitchison distances.

To further quantify the global similarity between 16S rRNA gene amplicon and WGS-derived microbial community structures, Procrustes analysis was performed using matched sample ordination coordinates from all 28 paired samples (Figure S1). Procrustes analysis revealed a high degree of concordance between the 16S rRNA and WGS sequencing platforms at both taxonomic levels. At the genus level, the two platforms showed strong similarity (Procrustes *m* ^*2*^ = 0.1464; *r* = 0.9239; *p* = 0.0001). While significant concordance was maintained at the species level (*r* = 0.8284, *p* = 0.0001), the higher residual sum of squares (*m* ^*2*^ = 0.3138) suggests increased platform-dependent divergence in community structure at finer taxonomic resolutions. Together, these results indicate that the two sequencing platforms captured significantly similar overall subgingival microbial community patterns, especially at the genus level.

### Platform-dependent differences in microbial profiles

**Figure 5.**
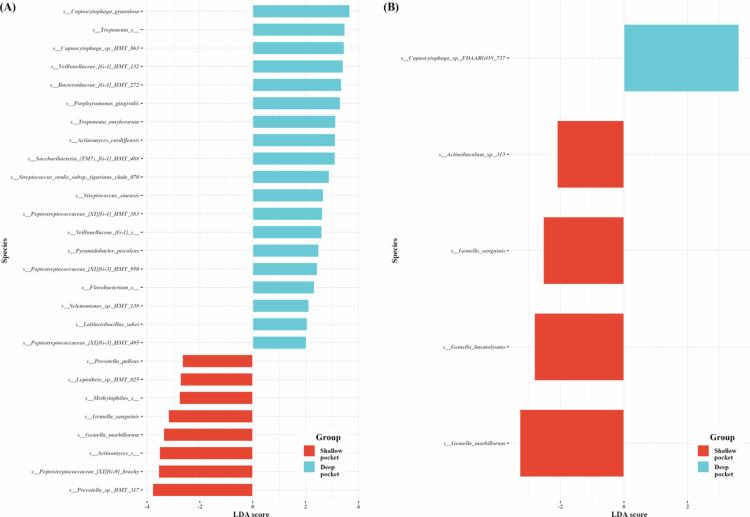
Comparisons of microbiota presented significantly different depending on the sequencing platforms. (A) 16S and (B) WGS. The analysis was performed using linear discriminant analysis (LDA) and effect size analysis. LDA score > 2.5 are displayed.

LEfSe analysis based on an LDA threshold of 2.5 revealed platform-dependent differences in identifying taxa significantly enriched between the shallow pocket and deep pocket (Figure 5). The 16S dataset identified 27 differentially enriched species, whereas the WGS dataset yielded 5. In the 16S dataset, taxa enriched in the deep pocket included *Porphyromonas gingivalis*, *Treponema spp., Capnocytophaga spp.,* and members of the *Saccharibacteria* (TM7) *spp.,* whereas the shallow pocket was enriched with taxa such as *Prevotella spp.* and *Gemella spp.* In contrast, the WGS dataset revealed enrichment of *Capnocytophaga spp.* in the deep pocket, while several *Gemella spp.* were enriched in the shallow pocket. Because microbiome relative-abundance data are compositional, additional compositionality-aware analyses were performed using centred log-ratio transformation and Aitchison distance. However, differential abundance analysis using MaAsLin2 or ALDEx2 identified significant taxa only in the 16S rRNA gene amplicon dataset, whereas no taxa remained significant in the WGS dataset after multiple-comparison correction (data not shown). Therefore, the LEfSe results were retained as exploratory analyses to illustrate platform-dependent differences in detected taxa.

### Platform-dependent differences in functional analysis

**Figure 6.**
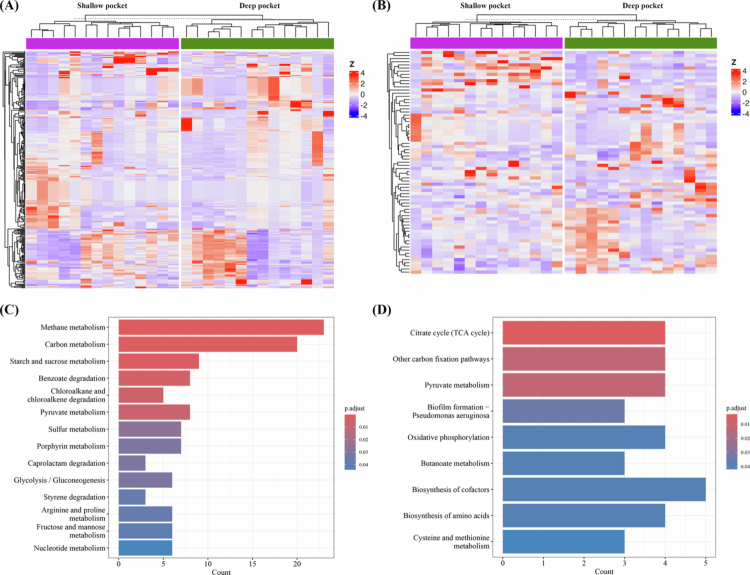
Functional profiling from 16S rRNA gene amplicon and WGS metagenomic data. (A, B) Heatmaps of significant KEGG Orthologs (KOs) identified from 16S-based PICRUSt2 prediction (A) and WGS-based HUMAnN profiling (B). Rows represent KOs, and the columns represent samples. The colour intensity indicates normalized KO abundance. Hierarchical clustering was performed based on Euclidean distance using complete linkage. Only KOs meeting the predefined significance criteria are displayed. (C, D) Summary of significantly enriched metabolic pathways inferred from the 16S-based PICRUSt2 analysis (C) and WGS-based HUMAnN analysis (D), highlighting shared and platform-specific functional patterns.

Following the taxonomic comparison, functional analyses were performed using platform-specific pipelines. For the 16S rRNA gene amplicon dataset, functional prediction was conducted using PICRUSt2, which infers KEGG Ortholog (KO) and pathway profiles based on reference genomes. For the WGS dataset, direct functional profiling was analyzed using HUMAnN. At the KO level, 6446 KOs were predicted by PICRUSt2 and a total of 326 significant KOs were identified in the 16S dataset, whereas 3595 KOs were profiled by HUMAnN and 71 significant KOs were detected in the WGS dataset (Figure 6A, B), showing a markedly higher number of significant KOs in the 16S-based analysis. At the pathway level, both analyses identified pyruvate metabolism and carbon-related pathways. However, the 16S-based PICRUSt2 prediction emphasized reductive and degradative pathways such as methane, sulphur and carbohydrate metabolism, whereas the WGS-based HUMAnN profiling highlighted oxidative and biosynthetic pathways, including oxidative phosphorylation and amino acid biosynthesis (Figure 6C, D).

As shown in Supplementary Table 2, the WGS dataset exhibited a wide range of microbial read rates. To evaluate the impact of this variability in sequencing output on KO detection, we examined the relationship between sequencing depth and functional profiling in the WGS dataset. Microbial (unmapped) read counts varied widely across samples, and samples with lower microbial read depth exhibited a substantially increased number of zero-count KOs (Figure S2A). When samples were regrouped by unmapped read counts of 10 million, samples with unmapped read counts of less than 10 million showed a significantly higher number of zero-count KOs (Figure S2B). Taken together, a lower microbial unmapped read count was associated with markedly more zero-count KOs, and samples with less than 10 million unmapped reads showed significantly inflated KO sparsity, indicating that insufficient microbial read depth can lead to underestimation of functional diversity in WGS-based profiling. In contrast, 16S rRNA gene amplicon sequencing maintained a stable proportion of non-chimeric reads across samples (Figure S3).

## Discussion

This study provides a direct comparison of 16S rRNA gene amplicon and WGS metagenomic sequencing using the same subgingival plaque samples from patients with periodontitis. Overall, our results demonstrate that although both platforms yield broadly comparable community profiles at higher taxonomic levels, they differ substantially in species-level composition, beta-diversity separation, differential abundance results and functional profiling. WGS provided higher taxonomic resolution, although beta-diversity differences between clinical groups were not statistically significant after compositionality-aware analysis. However, its performance was strongly affected by host-DNA contamination and large variability in microbial read depth, which resulted in sparse and unstable functional profiles and a reduced number of significant KOs in low-depth samples. In contrast, 16S rRNA gene amplicon sequencing produced more consistent sequencing output, more stable taxonomic profiles, and more statistically robust LEfSe and functional prediction results across samples.

Periodontitis is a chronic inflammatory disease of the supporting tissues of the teeth, driven by a dysbiotic subgingival microbiota and the host immune response [3,4]. The subgingival niche provides an anaerobic, protein-rich environment that supports colonization by fastidious Gram-negative bacteria [41,42]. The subgingival microbiota plays a central role in periodontitis by shifting from a symbiotic to a dysbiotic community, enabling immune subversion, driving chronic inflammation, and contributing to connective tissue and bone destruction [3,43]. Studying the subgingival microbiome is uniquely challenging owing to the limited raw biomass, high levels of host DNA contamination, strong site-to-site heterogeneity, and the dominance of anaerobic, disease-associated taxa. These features make it distinct from other oral niches (saliva, supragingival plaque and buccal mucosa) and gut microbiome studies, where the biomass is larger, contamination is lower, and community structure is more homogeneous [4,41,44]. While numerous studies have evaluated the differences between these sequencing approaches in the context of the human microbiome [15,26–29], direct comparative analyses in the oral microbiome remain scarce. Our findings fill this gap by providing a systematic comparison of platform-dependent differences in both taxonomic and functional outcomes.

WGS metagenomic sequencing, which captures the entire genomic DNA without targeted amplification, demonstrated significantly higher taxonomic resolution than 16S rRNA gene amplicon sequencing, which targets only selected hypervariable regions [14,20,21]. There are several differences between WGS and 16S rRNA gene amplicon sequencing: basic algorithm difference, reference database, and the number of sequence reads. As taxonomic resolution increased, the total number of detected taxa in WGS outpaced 16S rRNA gene amplicon sequencing substantially, while the proportion of overlapping taxa between the two platforms declined at higher taxonomic levels, such as genus and species, highlighting platform-specific detection biases (Figures 1–3). In terms of diversity, both platforms showed no significant differences in alpha and beta diversity between the shallow pocket and deep pocket (Figure 4). Although WGS provides higher taxonomic resolution than 16S rRNA gene amplicon sequencing, this increased resolution did not translate into statistically significant separation of overall community structure in the present dataset. The lack of significant beta-diversity separation between the shallow pocket and deep pocket should be interpreted with caution. Previous studies have reported differences in subgingival microbial composition between shallow and deep periodontal pockets, supporting the concept that increasing pocket depth is associated with ecological changes in the subgingival microbiome. However, these studies often compared more clearly separated clinical categories, such as shallow sites of ≤ 3 mm and deep sites of > 5 mm, and frequently used paired site-level sampling within the same individuals [52]. In contrast, our study compared groups with a relatively modest difference in PPD and included only patients with periodontitis, which may have reduced the contrast between groups. In addition, subgingival plaque is characterized by substantial inter-individual and site-level heterogeneity, and this variability may obscure pocket-depth-associated differences in global ordination analyses, particularly in a modest-sized cohort [53]. Therefore, the absence of significant beta-diversity separation in both 16S rRNA gene amplicon and WGS datasets does not necessarily indicate that pocket depth is unrelated to microbial dysbiosis. Rather, our results suggest that, in this cohort, pocket-depth-associated differences were not strong enough to produce a robust global community-level separation. Accordingly, taxon-level and functional differences should be interpreted as exploratory platform-dependent signals rather than as evidence of a definitive whole-community shift.

Distinct differences in detection patterns were observed between the two sequencing platforms. Bacteria more frequently detected by WGS predominantly belonged to the phylum Actinomycetota, including *Actinomyces*, *Corynebacterium*, *Olsenella* and *C. matruchotii*. Members of this phylum are characterized by relatively large genome sizes and high GC content, while often hindering PCR amplification [54]. Conversely, several taxa were more frequently detected by 16S rRNA gene amplicon sequencing, particularly Saccharibacteria (TM7). Members of this phylum are ultra-small bacteria with highly reduced genomes and a host-dependent parasitic lifestyle, for which targeted amplification of conserved 16S regions facilitates detection [41,55,56]. Similarly, low-abundance taxa such as *Granulicatella*, *Dialister*, *K. oralis* and *O. uli* were more abundantly represented in the 16S dataset.

Some taxa showed strong inter-platform correlations. Genera such as *Rothia*, *Cardiobacterium* and *Neisseria* represent components of the core oral microbiota. These taxa are generally characterized by high abundance, ecological stability, and taxonomically distinct marker genes, which underlie their reproducible detection across sequencing strategies [4,41,57]. The consistent detection of such taxa across platforms strengthens their potential utility as reliable biomarkers for oral health and disease. In contrast, other taxa showed weak inter-platform correlations, with notable examples including *Streptococcus*, *Prevotella* and *Porphyromonas*. For *Prevotella* and *Porphyromonas*, the high degree of species-level diversity and low inter-species correlation often observed within these genera means that genus-level aggregation hides ecological and abundance variability [57,58]. Furthermore, 16S rRNA gene amplicon sequencing has limited distinction for closely related species, and WGS, although offering higher resolution, can introduce noise through host DNA contamination and potential misclassification of low-abundance species [19,24,31,59]. Together, these biological and methodological factors likely underlie the inconsistencies observed between platforms. For *Streptococcus*, the correlation slope was broadly maintained, but the overall genus-level correlation appeared weak. *Streptococcus* is a core taxon in the oral microbiome with consistently high abundance [41,57], and because the differences between samples are relatively small, the overall range of variation is narrowed, leading to a weaker correlation. These observations highlight the importance of accounting for taxon-specific ecological and methodological characteristics when interpreting microbiome data across sequencing platforms.

When LEfSe was applied to identify taxa that are differentially enriched between groups based on relative abundance [51], the two platforms yielded notably different results. This discrepancy does not simply indicate a failure of WGS to detect certain taxa but likely reflects intrinsic differences in data characteristics. Specifically, 16S rRNA gene amplicon sequencing amplifies a conserved target gene, resulting in relatively uniform coverage across bacterial taxa and enabling the detection of even low-abundance species [60]. In contrast, WGS distributes reads across entire genomes, making detection more sensitive to genome size, complexity, and host DNA contamination, which may push low-abundance taxa below detection thresholds [23,61]. For example, several disease-associated taxa, including *P. gingivalis* and *Treponema spp.*, were identified as enriched in the deep pocket by 16S rRNA gene amplicon, while WGS highlighted a smaller set of taxa. Therefore, while 16S rRNA gene amplicon sequencing appears more suitable for LEfSe-based community-wide comparisons, WGS may be better suited for identifying specific taxa with functional or pathogenic relevance.

PICRUSt2 infers functional profiles from taxonomic compositions using reference genomes and phylogenetic placement, whereas HUMAnN directly quantifies gene families and pathways from WGS reads [48,49]. Thus, PICRUSt2 produces dense and stable functional matrices based on prediction, while HUMAnN provides biologically precise but sparsely populated profiles that depend heavily on microbial sequencing depth [49]. In Figure 6A, 16S-based PICRUSt2 prediction identified 326 significant KOs, whereas WGS-based HUMAnN detected only 71 significant KOs. WGS-based functional profiling is highly sensitive to the proportion of microbial reads retained after host DNA removal, and sample-to-sample variation in microbial read depth can substantially reduce pathway coverage [20,62]. Despite WGS generating far greater total read counts and offering higher taxonomic resolution, it yielded fewer significant KOs because the proportion of unmapped microbial reads varied dramatically across samples (2.75–91.91%). In our dataset, samples showed large variation in unmapped microbial read counts (Supplementary Table 2), and those with fewer unmapped reads exhibited markedly more zero-count KOs (Figure S2), indicating that uneven microbial read depth produces sparse functional matrices and limits KO detection. In contrast, 16S rRNA gene amplicon sequencing maintained a stable proportion of non-chimeric reads across samples, resulting in more uniform functional predictions and a larger number of significant KOs. Thus, although all samples were retained in this study to preserve direct paired comparisons across sequencing platforms, stabilizing microbial read depth remains a critical factor for ensuring the reliability of WGS-based functional analyses.

Similar issues have been noted in large-scale oral microbiome surveys, where host DNA contamination has been identified as a major technical barrier [63]. Several strategies can be considered to overcome this limitation. Methylation-based separation using methyl-CpG binding proteins can selectively remove human DNA while preserving microbial sequences [64]. Chemical approaches, such as osmotic lysis with propidium monoazide (lyPMA), have been shown to significantly reduce host-derived reads in oral metagenomes while minimizing taxonomic bias [65]. Commercial kits, including MolYsis and QIAamp Microbiome, offer additional options, but their efficiency and microbial yield on tissue samples must be carefully validated [66]. Finally, careful sampling procedures to minimize bleeding and epithelial shedding are essential, as emphasized in large-scale oral microbiome surveys [63]. Together, these strategies may help maximize the analytical value of WGS in periodontal research.

Periodontal inflammation is characterized by pronounced redox imbalance and excessive production of reactive oxygen species (ROS), which have long been recognized as key pathogenic mechanisms contributing to extracellular matrix degradation, osteoclast activation and alveolar bone resorption [67,68]. This concept has been consistently reinforced by recent studies, which increasingly indicate that oxidative stress and redox imbalance are not merely byproducts of inflammation, but also active biological processes that sustain chronic inflammation and shape a pathological tissue microenvironment [69,70]. In this context, it is noteworthy that the oxidative and biosynthetic pathways preferentially detected by WGS-based functional profiling are closely related to these key pathogenic mechanisms in periodontitis. Therefore, our findings suggest that WGS-based functional profiling can provide additional biological insight into the inflammatory and oxidative microenvironment of periodontal lesions, beyond their taxonomic composition.

There are several limitations to be considered. First, the analysis was limited to subgingival plaque samples from patients with periodontitis, which may restrict the generalizability of the findings to other oral niches or healthy individuals. Second, 16S rRNA gene amplicon sequencing targeted only the V1–V2 regions, and the use of different hypervariable regions might yield different microbial profiles. Third, although matched samples were analyzed using both methods, potential variability in library preprocessing may have biased the comparative results. Thus, future studies incorporating healthy cohorts and deeper sequencing approaches are warranted.

In summary, this study clarifies the methodological distinctions and points of convergence between 16S rRNA gene amplicon (V1–V2) sequencing and WGS metagenomic profiling in subgingival microbiome research. Despite these methodological differences, the two platforms shared several consistent taxonomic patterns and high-abundance functional pathways, suggesting that 16S rRNA gene amplicon (V1–V2) sequencing reliably captures the core features of the subgingival microbiome. In contrast, WGS enabled more detailed species-level and functional characterization and facilitated analyses related to oxidative processes, a key pathogenic feature of periodontitis; however, its performance was strongly dependent on achieving sufficient microbial read depth in the context of high host-DNA contamination. Our findings contribute to improving study design, data comparability, and the reproducibility of future oral microbiome research.

## Supplementary Material

Supplementary material Fig_S2_v1.tif

Supplementary material Fig_S3_v1.tif

Supplementary Material Fig_S1_procrust.tif

supplementary material xxxxxx.

## Data Availability

The raw sequencing data have been deposited in NCBI GenBank under BioProject ID PRJEB98488.
