# Supplementary figures and images for: Comparison of 16S rRNA gene amplicon and whole-genome shotgun metagenomic sequencing for subgingival oral microbiome profiling
Source: J Oral Microbiol. 2026 May 27;18(1):2679807. doi: 10.1080/20002297.2026.2679807 (PMC13218309; doi:10.1080/20002297.2026.2679807)

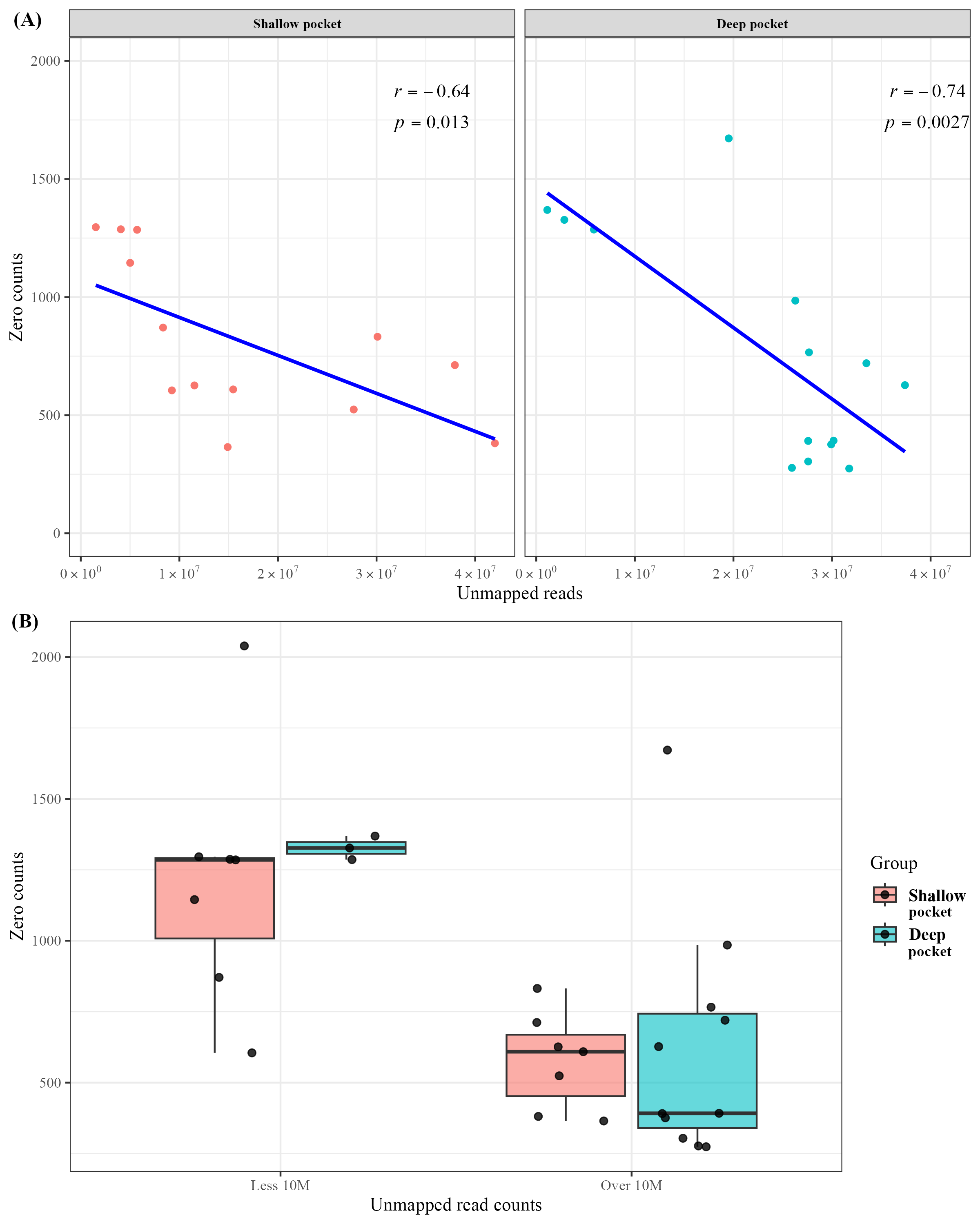

Supplement: Supplementary material — Fig_S2_v1.tif [file ZJOM_A_2679807_SM4362.tif]

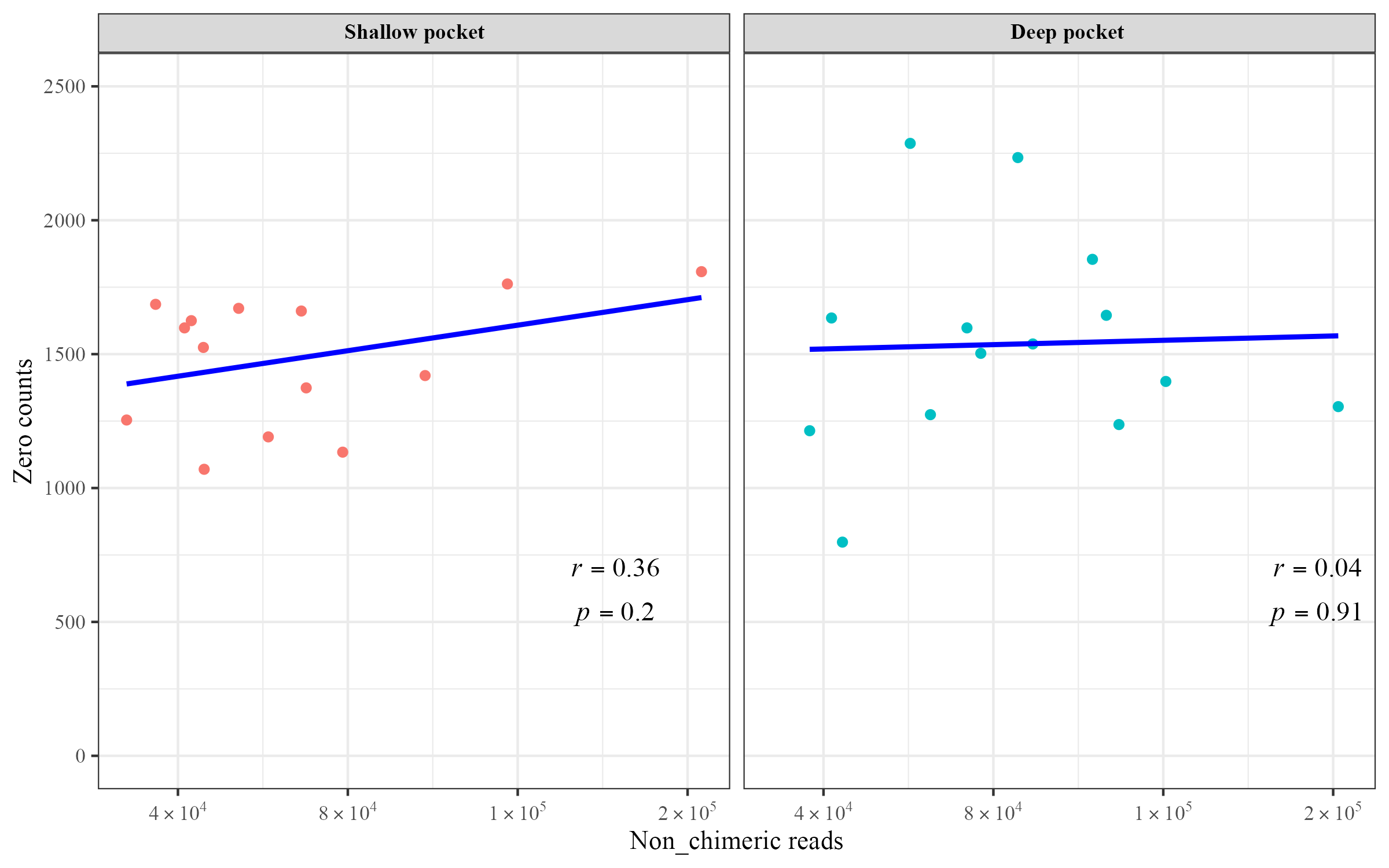

Supplement: Supplementary material — Fig_S3_v1.tif [file ZJOM_A_2679807_SM4363.tif]

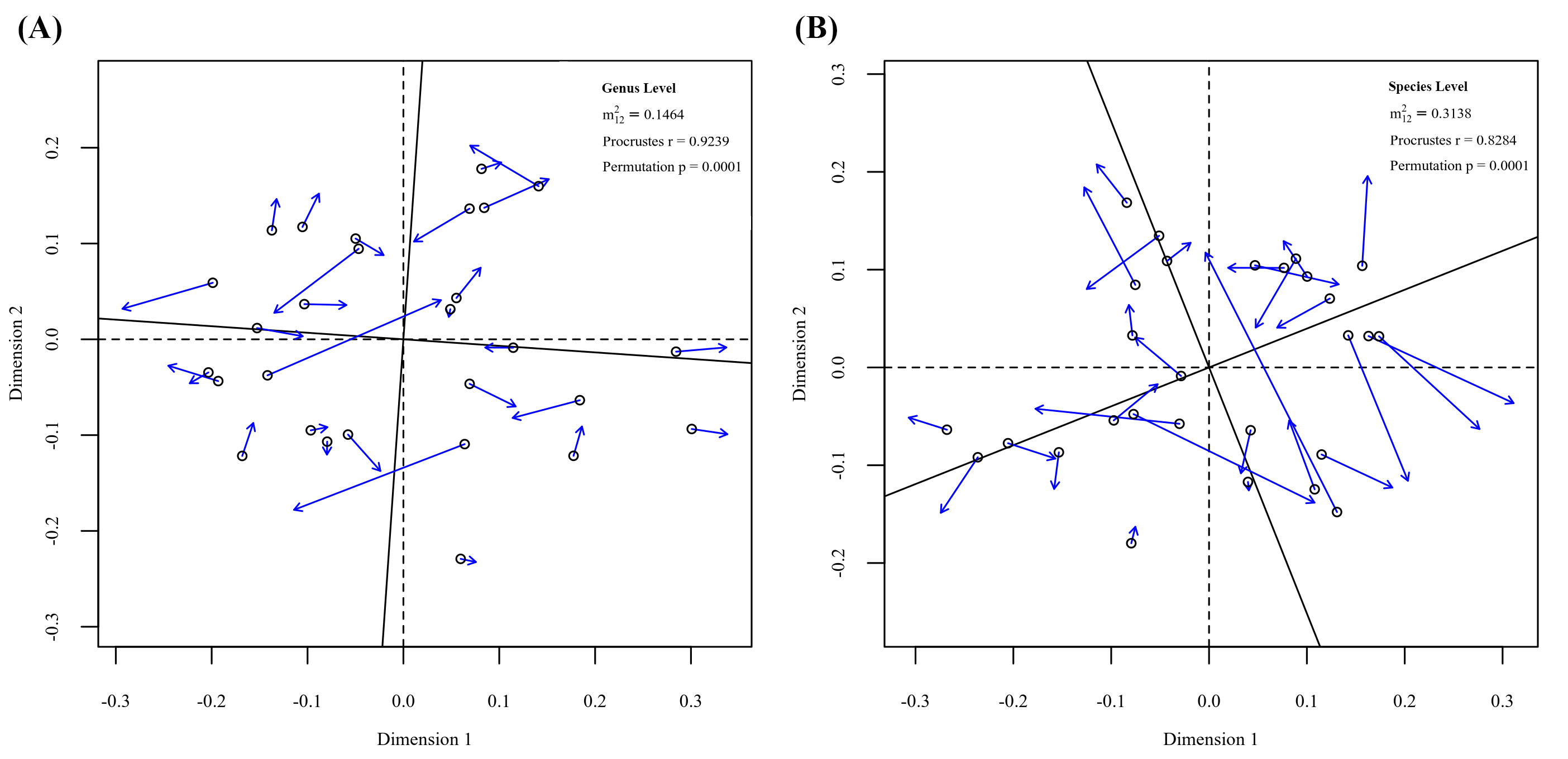

Supplement: Supplementary Material — Fig_S1_procrust.tif [file ZJOM_A_2679807_SM4361.tif]
