# Supplementary material for: Comparison of 16S rRNA gene amplicon and whole-genome shotgun metagenomic sequencing for subgingival oral microbiome profiling
Source: J Oral Microbiol. 2026 May 27;18(1):2679807. doi: 10.1080/20002297.2026.2679807 (PMC13218309; doi:10.1080/20002297.2026.2679807)
Supplement: supplementary material — xxxxxx. [file ZJOM_A_2679807_SM4796.docx]

**Supplementary Table 1. Summary of 16S amplicon data preprocessing**

| **sample** | **input** | **filtered** | **denoised** | **merged** | **non-chimeric** | **input non-chimeric (%)** |
| --- | --- | --- | --- | --- | --- | --- |
| Sample_1 | 121,022 | 105,924 | 103,428 | 94,281 | 41,884 | 34.61 |
| Sample_2 | 453,713 | 395,473 | 390,943 | 372,964 | 161,204 | 35.53 |
| Sample_3 | 229,327 | 201,807 | 198,885 | 186,948 | 73,778 | 32.17 |
| Sample_4 | 247,539 | 212,801 | 208,559 | 191,626 | 89,284 | 36.07 |
| Sample_5 | 232,808 | 203,711 | 200,893 | 189,552 | 77,032 | 33.09 |
| Sample_6 | 122,803 | 92,511 | 90,155 | 82,298 | 36,743 | 29.92 |
| Sample_7 | 121,720 | 106,732 | 104,577 | 97,924 | 41,546 | 34.13 |
| Sample_8 | 317,965 | 276,442 | 271,535 | 249,424 | 103,320 | 32.49 |
| Sample_9 | 377,454 | 331,151 | 325,403 | 297,833 | 109,563 | 29.03 |
| Sample_10 | 375,051 | 338,244 | 334,995 | 315,805 | 106,589 | 28.42 |
| Sample_11 | 398,597 | 347,721 | 341,376 | 316,346 | 120,600 | 30.26 |
| Sample_12 | 281,242 | 244,557 | 239,963 | 221,724 | 85,755 | 30.49 |
| Sample_13 | 572,336 | 503,641 | 496,633 | 464,954 | 163,268 | 28.53 |
| Sample_14 | 146,102 | 127,169 | 124,517 | 115,880 | 45,993 | 31.48 |
| Sample_15 | 372,420 | 318,728 | 312,631 | 287,947 | 117,571 | 31.57 |
| Sample_16 | 150,283 | 133,288 | 130,887 | 122,257 | 46,176 | 30.73 |
| Sample_17 | 231,370 | 205,502 | 201,021 | 184,147 | 70,202 | 30.34 |
| Sample_18 | 185,427 | 163,480 | 160,108 | 148,604 | 54,294 | 29.28 |
| Sample_19 | 108,181 | 87,873 | 85,548 | 78,523 | 27,914 | 25.8 |
| Sample_20 | 108,890 | 89,484 | 87,469 | 81,775 | 34,740 | 31.9 |
| Sample_21 | 214,989 | 179,330 | 175,553 | 164,855 | 61,276 | 28.5 |
| Sample_22 | 202,876 | 178,535 | 175,473 | 165,302 | 69,052 | 34.04 |
| Sample_23 | 121,959 | 108,949 | 106,677 | 97,142 | 44,462 | 36.46 |
| Sample_24 | 203,705 | 172,425 | 169,034 | 155,777 | 65,174 | 31.99 |
| Sample_25 | 238,467 | 213,584 | 209,630 | 195,362 | 78,793 | 33.04 |
| Sample_26 | 298,525 | 258,228 | 254,381 | 238,404 | 98,195 | 32.89 |
| Sample_27 | 175,534 | 154,656 | 151,325 | 140,090 | 60,414 | 34.42 |
| Sample_28 | 119,541 | 104,265 | 102,157 | 92,864 | 43,143 | 36.09 |
| average | 240,352 | 209,150 | 205,491 | 191,093 | 75,999 | 31.9 |
| std | 118,580 | 105,065 | 103,853 | 97,591 | 36,045 | 2.7 |

**Supplementary Table 2. Summary of WGS data preprocessing**

| **Sample** | **input** | **trimmomatrics** | **Bowtie** | | **Trimmed**  **(%)** | **Bowtie (%)** | |
| --- | --- | --- | --- | --- | --- | --- | --- |
|  |  |  | **unmapped** | **Host_mapped** |  | **Unmapped** | **Host mapped** |
| Sample_1 | 41,594,486 | 38,290,776 | 37,940,456 | 350,320 | 92.06% | 91.22% | 0.84% |
| Sample_2 | 29,853,826 | 27,106,728 | 1,123,808 | 25,982,920 | 90.80% | 3.76% | 87.03% |
| Sample_3 | 38,150,598 | 34,936,388 | 5,849,094 | 29,087,294 | 91.57% | 15.33% | 76.24% |
| Sample_4 | 45,700,930 | 42,284,360 | 42,004,378 | 279,982 | 92.52% | 91.91% | 0.61% |
| Sample_5 | 33,040,804 | 30,304,792 | 30,096,714 | 208,078 | 91.72% | 91.09% | 0.63% |
| Sample_6 | 31,925,504 | 29,584,310 | 25,932,304 | 3,652,006 | 92.67% | 81.23% | 11.44% |
| Sample_7 | 30,675,296 | 27,990,802 | 5,010,174 | 22,980,628 | 91.25% | 16.33% | 74.92% |
| Sample_8 | 37,959,722 | 34,856,578 | 33,484,080 | 1,372,498 | 91.83% | 88.21% | 3.62% |
| Sample_9 | 33,091,144 | 30,373,690 | 27,579,298 | 2,794,392 | 91.79% | 83.34% | 8.44% |
| Sample_10 | 30,624,478 | 27,968,126 | 27,664,974 | 303,152 | 91.33% | 90.34% | 0.99% |
| Sample_11 | 40,967,210 | 37,674,144 | 19,522,766 | 18,151,378 | 91.96% | 47.65% | 44.31% |
| Sample_12 | 34,586,824 | 31,900,582 | 31,741,732 | 158,850 | 92.23% | 91.77% | 0.46% |
| Sample_13 | 42,108,020 | 38,885,950 | 37,396,294 | 1,489,656 | 92.35% | 88.81% | 3.54% |
| Sample_14 | 32,091,984 | 29,072,294 | 14,898,388 | 14,173,906 | 90.59% | 46.42% | 44.17% |
| Sample_15 | 34,368,148 | 31,549,528 | 26,274,392 | 5,275,136 | 91.80% | 76.45% | 15.35% |
| Sample_16 | 34,802,612 | 32,040,868 | 30,155,522 | 1,885,346 | 92.06% | 86.65% | 5.42% |
| Sample_17 | 41,447,540 | 37,540,530 | 2,850,412 | 34,690,118 | 90.57% | 6.88% | 83.70% |
| Sample_18 | 30,157,896 | 27,647,328 | 27,580,062 | 67,266 | 91.68% | 91.45% | 0.22% |
| Sample_19 | 32,665,286 | 30,010,026 | 29,913,554 | 96,472 | 91.87% | 91.58% | 0.30% |
| Sample_20 | 32,244,606 | 29,359,368 | 4,066,292 | 25,293,076 | 91.05% | 12.61% | 78.44% |
| Sample_21 | 32,398,348 | 29,366,532 | 9,243,394 | 20,123,138 | 90.64% | 28.53% | 62.11% |
| Sample_22 | 42,790,500 | 38,742,560 | 8,346,768 | 30,395,792 | 90.54% | 19.51% | 71.03% |
| Sample_23 | 31,578,588 | 28,638,304 | 869,528 | 27,768,776 | 90.69% | 2.75% | 87.94% |
| Sample_24 | 34,375,622 | 31,439,066 | 11,526,182 | 19,912,884 | 91.46% | 33.53% | 57.93% |
| Sample_25 | 34,244,628 | 31,219,398 | 15,456,058 | 15,763,340 | 91.17% | 45.13% | 46.03% |
| Sample_26 | 32,086,006 | 29,173,978 | 5,713,428 | 23,460,550 | 90.92% | 17.81% | 73.12% |
| Sample_27 | 40,869,500 | 37,512,590 | 27,682,840 | 9,829,750 | 91.79% | 67.73% | 24.05% |
| Sample_28 | 35,039,636 | 31,652,546 | 1,522,850 | 30,129,696 | 90.33% | 4.35% | 85.99% |
| Average | 35,408,562 | 32,397,219 | 19,337,348 | 13,059,871 | 91.47% | 54.01% | 37.46% |
| SD | 4,511,727 | 4,205,053 | 13,105,860 | 12,177,549 | 0.66% | 35.33% | 34.81% |

**Supplementary Figure legends**

Figure S1. Procrustes analysis comparing 16S rRNA gene amplicon and WGS metagenomic community structures. (A) Genus-level Procrustes analysis and (B) species-level Procrustes analysis were performed using ordination coordinates derived from Aitchison distances calculated from CLR-transformed abundance profiles. Each point represents a matched subgingival plaque sample, and arrows connect the corresponding 16S and WGS ordination positions after Procrustes rotation. Shorter arrows indicate higher concordance between the two platforms for a given sample. The analysis showed significant similarity between the two sequencing platforms (Procrustes correlation = 0.8284, m12² = 0.3138, permutation p = 0.0001; 9,999 permutations).

Figure S2. Association between unmapped reads and KO zero-count across groups in WGS data. (A) Correlation between the number of unmapped reads and the number of zero-count KOs per sample, stratified by group. Blue lines indicate linear regression fits for each group. The Pearson correlation coefficient (r) and corresponding p value are displayed in each panel. (B) Number of zero KOs counts when samples were stratified by unmapped read counts using 10 million reads as the cutoff.

Figure S3. Association between unmapped reads and KO zero-count across groups in 16S data. (A) Scatter plots of the correlation between the number of unmapped reads and the number of zero-count KOs per sample, stratified by group. Blue lines indicate linear regression fits for each group. The Pearson correlation coefficient (r) and corresponding p value are displayed in each panel.
